# Novel *GTF2I*–*PDGFRB* and *IKZF1*–*TYW1* fusions in pediatric leukemia with normal karyotype

**DOI:** 10.1186/s40164-019-0136-y

**Published:** 2019-05-29

**Authors:** Ioannis Panagopoulos, Marta Brunetti, Margrethe Stoltenberg, Rønnaug A. U. Strandabø, Julie Staurseth, Kristin Andersen, Ilyá Kostolomov, Tarjei S. Hveem, Susanne Lorenz, Tove Anita Nystad, Trond Flægstad, Francesca Micci, Sverre Heim

**Affiliations:** 1 Section for Cancer Cytogenetics, Institute for Cancer Genetics and Informatics, The Norwegian Radium Hospital, Oslo University Hospital, Montebello, Nydalen, PO Box 49534, 0424 Oslo, Norway; 2 Section for Applied Informatics, Institute for Cancer Genetics and Informatics, The Norwegian Radium Hospital, Oslo University Hospital, Oslo, Norway; 3 Genomics Core Facility, Department of Core Facilities, Oslo University Hospital, Oslo, Norway; 4 Department of Pediatrics, Division of Child and Adolescent Health, University Hospital of North-Norway, 9038 Tromsø, Norway; 5 Pediatric Research Group, Department of Clinical Medicine, Faculty of Health Science, The Arctic University of Norway-UiT, 9037 Tromsø, Norway; 6 Institute of Clinical Medicine, Faculty of Medicine, University of Oslo, Oslo, Norway

**Keywords:** Pediatric acute lymphoblastic leukemia, Normal karyotype, Submicroscopic deletions, Fusion genes, *GTF2I*–*PDGFRB*, Fluorescence in situ hybridization, Array comparative genomic hybridization, RNA sequencing, *GTF2I*–*PDGFRB*, *IKZF1*–*TYW1*

## Abstract

**Background:**

Many cases of acute lymphoblastic leukemia (ALL) carry visible acquired chromosomal changes of pathogenetic, diagnostic, and prognostic importance. Nevertheless, from one-fourth to half of newly diagnosed ALL patients have no visible chromosomal changes detectable by G-banding analysis at diagnosis. The introduction of powerful molecular methodologies has shown that many karyotypically normal ALLs carry clinically important submicroscopic aberrations.

**Case presentation:**

We used fluorescence in situ hybridization (FISH), array comparative genomic hybridization (aCGH), RNA sequencing, reverse transcription (RT) and genomic polymerase chain reaction (PCR), as well as Sanger sequencing to investigate a case of pediatric ALL with a normal karyotype. FISH with a commercial *PDGFRB* breakapart probe showed loss of the distal part of the probe suggesting a breakpoint within the *PDGFRB* locus. aCGH revealed submicroscopic deletions in chromosome bands 5q32q35.3 (about 30 Mb long, starting within *PDGFRB* and finishing in the *CANX* locus), 7q34 (within *TCRB*), 9p13 (*PAX5*), 10q26.13 (*DMBT1*), 14q11.2 (*TRAC*), and 14q32.33 (within the *IGH* locus). RNA sequencing detected an in-frame *GTF2I*–*PDGFRB* and an out-of-frame *IKZF1*–*TYW1* fusion transcript. Both fusion transcripts were verified by RT-PCR together with Sanger sequencing and interphase FISH. The *GTF2I*–*PDGFRB* fusion was also verified by genomic PCR and FISH. The corresponding *GTF2I*–*PDGFRB* fusion protein would consist of almost the entire GTF2I and that part of PDGFRB which harbors the catalytic domain of the tyrosine kinase. It would therefore seem to lead to abnormal tyrosine kinase activity in a manner similar to what has been seen for other PDGFRB fusion proteins.

**Conclusions:**

The examined pediatric leukemia is a Ph-like ALL which carries novel *GTF2I*–*PDGFRB* and *IKZF1*–*TYW1* fusion genes together with additional submicroscopic deletions. Because hematologic neoplasms with *PDGFRB*-fusion genes can be treated with tyrosine kinase inhibitors, the detection of such novel fusions may be clinically important. Since the *GTF2I*–*PDGFRB* could be detected only after molecular studies of the leukemic cells, further investigations of ALL-cases, perhaps especially but not exclusively with a normal karyotype, are needed in order to determine the frequency of *GTF2I*–*PDGFRB* in leukemia, and also to find out which clinical impact the fusion may have.

## Background

Most cases of acute lymphoblastic leukemia (ALL) carry visible acquired chromosomal changes of pathogenetic, diagnostic, and prognostic importance [1]. However, up to 43% of newly diagnosed ALL patients are reported to carry normal G-banded karyotypes at diagnosis [2–4]. The introduction of powerful molecular methodologies such as fluorescence in situ hybridization (FISH), array based gene expression analysis of mRNA and miRNA, array comparative genomic hybridization (aCGH), single nucleotide polymorphism (SNP) arrays, and, very recently, whole genome sequencing, whole-exome sequencing, and RNA sequencing, has shown that many of the karyotypically normal ALLs nevertheless carry clinically important submicroscopic genetic aberrations [2, 5–9]. Using SNP methodology, Paulsson et al. [6] showed that almost all 45 examined adult and adolescent ALL cases carried cryptic genetic changes. Similar results were obtained by Okamoto et al. [7] who studied with SNP methodology 75 adult ALLs and compared them with 399 pediatric ALLs, and by Othman et al. [8] who studied 61 karyotypically normal ALL cases. A common theme in the published studies is that deletions of genes involved in B lymphopoiesis and cell-cycle regulation, such as *CDKN2A*, *EBF1, ETV6*, *IKZF1*, *PAX5*, and *RB1*, occur with a high frequency [6–8].

In the present study, we used FISH, aCGH, and RNA sequencing to further examine a karyotypically normal case of pediatric ALL finding novel fusion genes and submicroscopic deletions.

## Case presentation

### Case report

The patient was a 3 years old girl who for 2 months had symptoms and signs of disease with fever and reduced general condition. The last week she had nose bleedings, petechiae, and pain from the throat and abdomen. The initial blood tests showed hemoglobin 4.3 g/dL (normal 11–14 g/dL), leukocytes 72 × 10^9^/L (normal 4 × 10^9^/L–15 × 10^9^/L), and thrombocytes 19 × 10^9^/L (normal 150 × 10^9^/L–450 × 10^9^/L). The blood and bone marrow smears revealed that the patient had acute lymphoblastic leukemia. Immunophenotyping of peripheral blood showed 80% pre-B-lymphoblasts, in the bone marrow 88%. The patient was treated according to the NOPHO ALL 2008 protocol, high-risk group [10]. She started with induction treatment, but the bone marrow on day 15 showed 90% lymphoblasts. The treatment was therefore changed to block treatment according to the protocol, and on day 34 minimal residual disease (MRD) was less than 0.01%. Also repeated later measurements have shown MRD of less than 0.01%.

### Genetic analyses

**Fig. 1:**
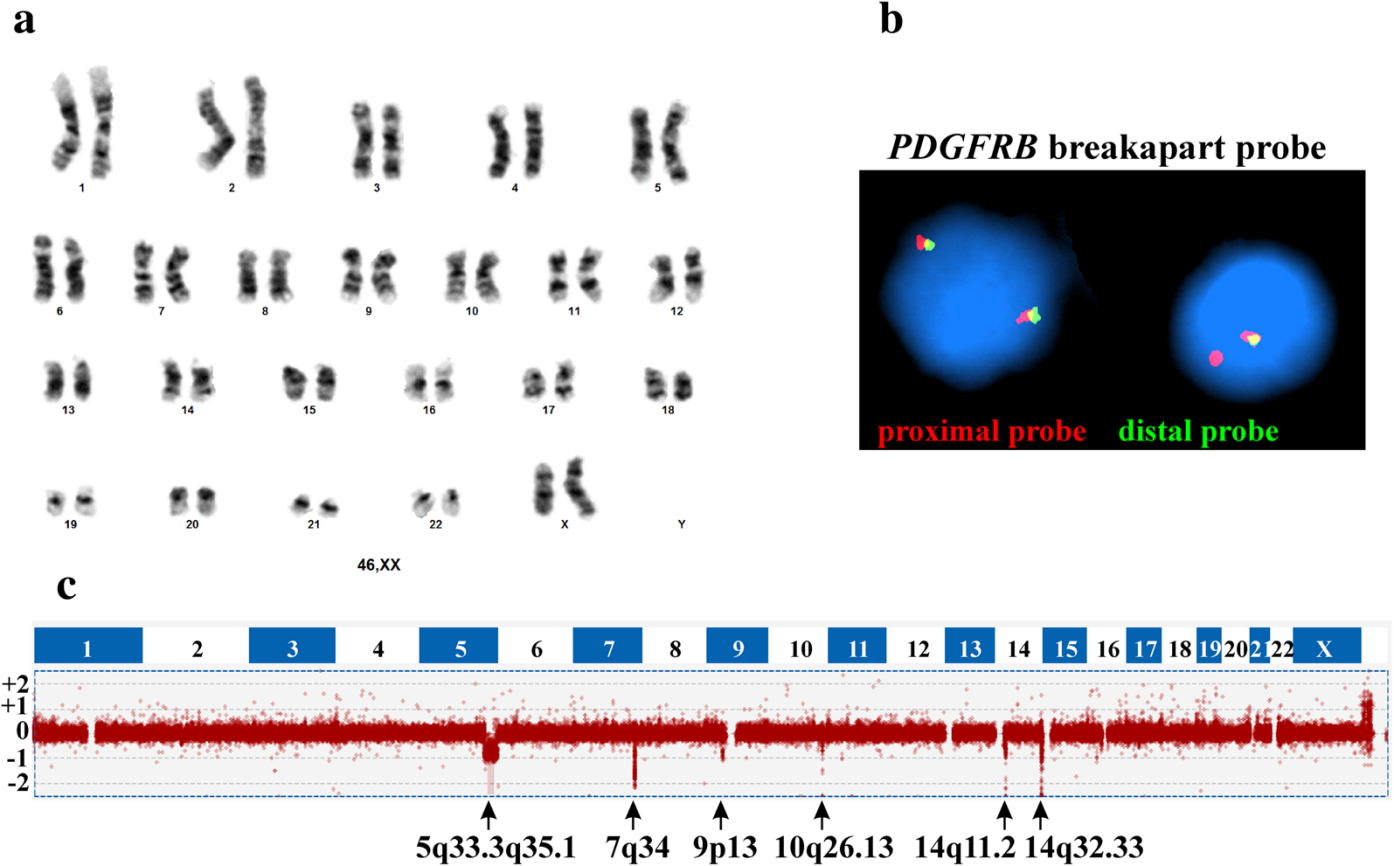
G-banding, FISH, and aCGH analyses. **a** The G-banding analysis showed a normal karyotype, 46,XX. **b** Interphase FISH with the *PDGFRB* breakapart probe on a normal nucleus and on a nucleus with loss of the distal probe suggesting a genetic breakpoint in the *PDGFRB* locus. **c** aCGH showing deletions on chromosome bands 5q32q35.3, 7q34, 9p13, 10q26.13, 14q11.2, and 14q32.33

The G-banding analysis at diagnosis of bone marrow and blood metaphase cells revealed a normal karyotype, 46,XX, in all 25 examined metaphases (Fig. 1a). Interphase FISH analyses with the Cytocell (Cytocell, Banbury, Oxfordshire, UK) multiprobe ALL panel did not detect aberrations of *MYC*, *CDKN2A, TCF3*, *MLL,* and *IGH,* no *ETV6*-*RUNX1* or *BCR*-*ABL1* fusions, nor was hyperdiploidy seen in 200 examined nuclei (data not shown). FISH with the *PDGFRB* breakapart probe (Cytocell) showed loss of the distal part of the probe in 176 out of 201 examined interphase nuclei from white blood cells and 42 out of 100 examined interphase nuclei from bone marrow cells, suggesting a genetic breakpoint in the *PDGFRB* locus in 5q32 (Fig. 1b).

aCGH was performed with genomic DNA extracted from the patientʼs peripheral blood cells using the Maxwell 16 Instrument System and the Maxwell 16 Cell DNA Purification Kit (Promega, Madison, USA). Promegaʼs human genomic female DNA (Promega, Madison, USA) was used as reference DNA. For aCGH, the CytoSure array products were used (Oxford Gene Technology, Begbroke, Oxfordshire, UK) following the company’s protocols. The CytoSure Genomic DNA Labelling Kit was used for labelling of one μg of patient’s and reference DNA, the CytoSure Cancer +SNP array was used for hybridization, and the CytoSure Interpret analysis software was used to analyse the results.

aCGH revealed submicroscopic deletions in chromosome bands 5q32q35.3, 7q34 (within *TCRB*), 9p13 (*PAX5*), 10q26.13 (*DMBT1*), 14q11.2 (*TRAC*), and 14q32.33 (within the *IGH* locus) (Fig. 1c, Table 1). The deletion on 5q was 30 Mb long, started between exons 8 and 9 of *PDGFRB* (5q32), and finished in the *CANX* locus (5q35.3). The result was in agreement with the FISH data obtained with the *PDGFRB* breakapart probe (Fig. 1b, c). Because both FISH and aCGH findings indicated a possible *PDGFRB*-fusion gene, one µg of the total RNA, extracted from the patient´s bone marrow at the time of diagnosis using miRNeasy Mini Kit (Qiagen Nordic, Oslo, Norway), was sent to the Genomics Core Facility at the Norwegian Radium Hospital, Oslo University Hospital (http://genomics.no/oslo/) for high-throughput paired-end RNA-sequencing. For library preparation from total RNA the Illumina TruSeq RNA Access Library Prep kit was used according to Illuminaʼs protocol (Illumina, San Diego, CA, USA; https://support.illumina.com/content/dam/illumina-support/documents/documentation/chemistry_documentation/samplepreps_truseq/truseqrnaaccess/truseq-rna-access-library-prep-guide-15049525-b.pdf). Sequencing was performed on NextSeq 550 System (Illumina) and 16 million reads were generated.

**Table 1:** The results of aCGH obtained in the pediatric ALL with normal karyotype and are based on Human, February 2009, GRCh37/hg19 assembly

Cytogenetic location	Start	Stop	Size	Imbalances
5q32q35.3	149511500	179127000	29.62 Mb	Loss
7q34	142004630	142500344	495.7 Kb	Loss
9p13.2	36929656	37030664	101.01 Kb	Loss
10q26.13	124339069	124380607	41.54 Kb	Loss
14q11.2	22691684	22951081	260 Kb	Loss
14q32.33	106167466	107240718	1.07 Mb	Loss

**Fig. 2:**
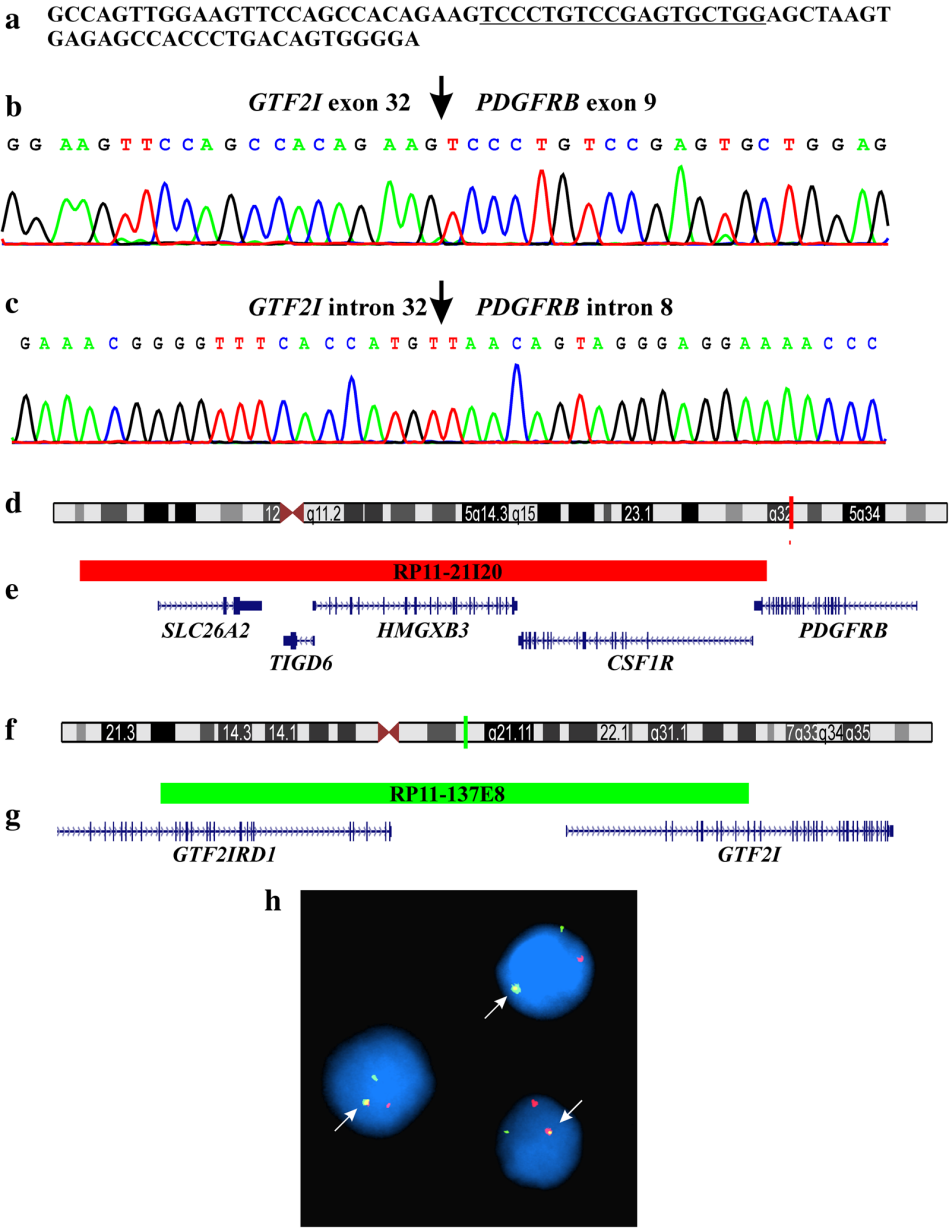
Molecular genetic and FISH analyses for identification of the *GTF2I*–*PDGFRB* fusion gene. **a** The 76 bp sequence obtained from the raw data of RNA sequencing using the command “grep”. The search term “TCCCTGTCCGAGTGCTGG” is underlined. **b** Partial sequence chromatogram of the PCR amplified cDNA fragment showing the fusion (arrow) of *GTF2I* and *PDGFRB*. **c** Partial sequence chromatogram of the PCR amplified genomic DNA fragment showing the fusion (arrow) of *GTF2I* and *PDGFRB.* The identification of the *GTF2I* gene as the *PDGFRB*-fusion partner was possible only after FISH experiments using appropriate commercial BAC probes for *PDGFRB* and *GTF2I*. **d** Ideogram of chromosome 5 showing the mapping position of the *PDGFRB* gene (vertical red line). **e** Diagram showing the FISH probe RP11-21I20 for *PDGFRB*. Additional genes in this region are also shown. **f** Ideogram of chromosome 7 showing the mapping position of the *GTF2I* gene (vertical green line). **g** Diagram showing the FISH probe RP11-137E8 for *GTF2I.* Additional genes in this region are also shown. **h** FISH on interphase nuclei with the *PDGFRB* (red signal) and *GTF2I* (green signal) probes showing a red signal, a green signal and one yellow-fusion signal (arrow)

Because the raw fastq RNA sequencing data were in the text-based format, we used the “grep” command-line utility to search for sequences which contained part of the ninth exon of *PDGFRB* [11]. Using the search term “TCCCTGTCCGAGTGCTGG”, which corresponds to 1713–1730 nt in the *PDGFRB* reference sequence with accession number NM_002609.3, only one 76 bp long sequence was extracted (Fig. 2a). BLAT of this sequence on the human genome browser-hg19 assembly (http://genome-euro.ucsc.edu/cgi-bin/hgGateway) showed that the sequence between nucleotides 26–76 mapped on chromosome 5 at position 149510177–149510227 and was part of exon 9 of *PDGFRB.* The sequence between nucleotides 1–27 (GCCAGTTGGAAGTTCCAGCCACAGAAG) mapped on chromosome 7 at three different positions: (a) chr7:74172307–74172333 (exon 32 of general transcription factor Iii, *GTF2I,* reference sequence: NM_032999.3), (b) chr7: 74603796–74603822 (exon 22 of general transcription factor IIi pseudogene 1, *GTF2IP1*, reference sequence: NR_002206.3), and (c) chr7:72618618–72618644 (exon 22 of general transcription factor IIi pseudogene 4, *GTF2IP4*, reference sequence: NR_003580.2). These data were verified when we used the BLAST algorithm (http://blast.ncbi.nlm.nih.gov/Blast.cgi) to compare the sequence with the reference sequences NM_002609.3 (*PDGFRB)*, NM_032999.3 (*GTF2I*), NR_002206.3 (*GTF2IP1*), and NR_003580.2 (*GTF2IP4)*.

In order to confirm the existence of the *GTF2I*–*PDGFRB* fusion gene, reverse transcription (RT) and genomic PCR analyses were performed as previously described [12]. The primers used for PCR amplifications and Sanger sequencing analyses are shown in Table 2. RT-PCR with the primers GTF2I-3306F1/PDGFRB-1732R1 amplified an 84 bp long cDNA fragment. Sanger sequencing of the PCR products verified the fusion which was found upon searching the RNA sequencing data using the “grep” command (Fig. 2b). Thus, the leukemic cells carried either the fusion transcript *GTF2IP1*-*PDGFRB*, or *GTF2I*–*PDGFRB*, or *GTF2IP4*-*PDGFRB*. Genomic PCR with the primers GTF2I-3317F1 and PDGFRB-1737R1 amplified a single 1200 bp fragment which by Sanger sequencing was shown to be a hybrid genomic DNA fragment in which intron 8 of *PDGFRB* is fused with either intron 22 of *GTF2IP1,* intron 32 of *GTF2I,* or intron 22 of *GTF2IP4* (Fig. 2c). Additional interphase FISH experiments were performed to detect the *GTF2I*–*PDGFRB* fusion gene (Fig. 2d–h). BACs RP11-21I20 and RP11-137E8 were retrieved from the Human “32K” BAC Re-Array library (BACPAC Resources, https://bacpacresources.org/home.htm). RP11-21I20, the probe for the *PDGFRB* gene, mapped to band 5q32 (Position: chr5: 149,320,375–149,496,703; UCSC Genome Browser on Human February 2009 GRCh37/hg19 Assembly) and was labeled red (Fig. 2d, e). RP11-137E8, the probe for the *GTF2I* gene, mapped to band 7q11.23 (Position: chr7: 73,944,720–74,129,587) and was labeled green (Fig. 2f, g). Detailed information about the FISH procedure was given previously [13]. Fluorescent signals were captured and analyzed using the CytoVision system (Leica Biosystems, Newcastle, UK). FISH analysis showed a fusion signal in 44 out of 100 examined interphase nuclei from bone marrow cells suggesting a *GTF2I*–*PDGFRB* fusion gene (Fig. 2h). Thus, FISH with specific probes for *PDGFRB* and *GTF2I* was crucial to show that a novel *GTF2I*–*PDGFRB* fusion gene had been formed (Fig. 2d–h).

**Table 2:** Primers used for PCR amplification and Sanger sequencing analyses

Name	Sequence (5′– > 3′)	Position	Reference sequence	Gene
GTF2I-3306F1	AATCAGCTGAACCAAGCCAGTTG	3306–3328	NM_032999.3	*GTF2I*
PDGFRB-1732R1	TGTCAGGGTGGCTCTCACTTAGC	1732–1754	NM_002609.3	*PDGFRB*
GTF2I-3317F1	CCAAGCCAG TTGGAAGTTCCAGCCA	3317–3341	NM_032999.3	*GTF2I*
PDGFRB-1737R1	TCCCCACTGTCAGGGTGGCTCTCAC	1737–1761	NM_002609.3	*PDGFRB*
IKZF1-469F1	GAATGCTTGATGCCTCGGGAGA	469–490	NM_006060.6	*IKZF1*
TYW1-1282R1	CCGAGTGGCTCCCAATCAACTG	1282–1303	NM_018264.3	*TYW1*

**Fig. 3:**
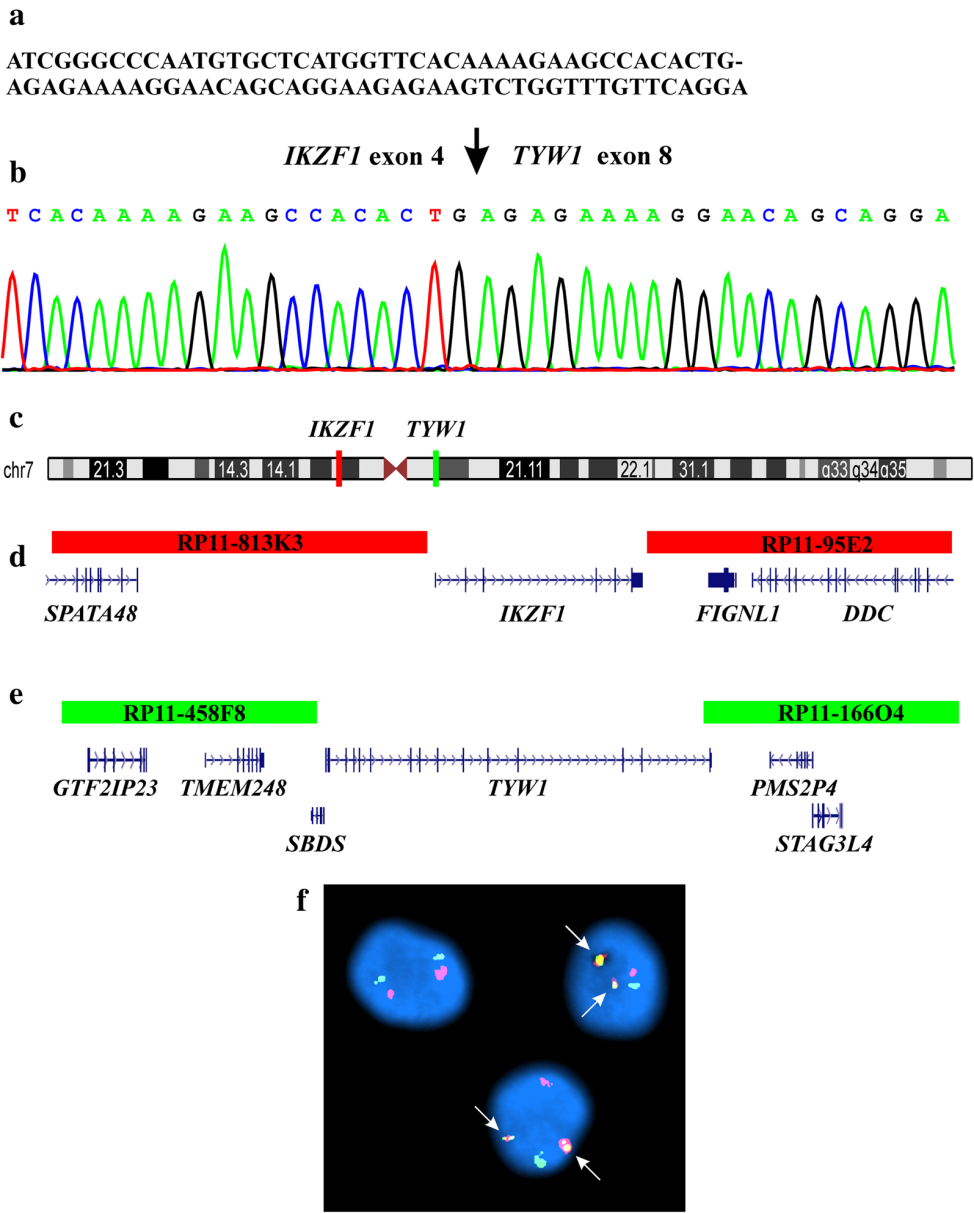
Molecular genetic and FISH analyses for identification of the *IKZF1*–*TYW1* fusion gene. **a** The sequence which was obtained using the FusionCatcher software with the fastq files of the RNA sequencing data. **b** Partial sequence chromatogram of the PCR amplified cDNA fragment showing the fusion (arrow) of *IKZF1* and *TYW1*. **c** Ideogram of chromosome 7 showing the mapping position of *IKZF1* (vertical red line) and *TYW1* (vertical green line). **d** Diagram showing the FISH probe (RP11-813K3 and RP11-95E2) for *IKZF1*. Additional genes in this region are also shown. **e** Diagram showing the FISH probe (RP11-458F8 and RP11-166O4) for *TYW1.* Additional genes in this region are also shown. **f** FISH on interphase nuclei with the *IKZF1* (red signal) and *TYW1* (green signal) probes showing a red signal, a green signal, and two yellow-fusion signals (arrow)

Using the FusionCatcher software [14] with the fastq files of the RNA sequencing data, an out-of-frame *IKZF1*–*TYW1* fusion transcript was found (Fig. 3a).

RT-PCR with the primers IKZF1-469F1/TYW1-1282R1 (Table 2) amplified a 319 bp long cDNA fragment which by Sanger sequencing was shown to contain *IKZF1*–*TYW1* (Fig. 3b). The fusion point thus detected was identical to that found by analysis of the RNA sequencing data using the FusionCatcher software (Fig. 3a, b). In the *IKZF1*–*TYW1* transcript, exon 4 of *IKZF1* (nt 642 in sequence with accession number NM_006060 version 6) was fused out-of-frame to exon 8 of *TYW1* (nt 1131 in NM_018264 version 4) (Fig. 3b).

The *IKZF1*–*TYW1* fusion gene would encode a putative truncated 159 aa IKZF1 protein containing the first 140 aa of IKZF1 (NP_006051) and 19 aa from the fused TYW1. This protein would not contain the functional domains of the normal IKZF1 protein. Alterations of *IKZF1* (often deletions) are strongly associated with *BCR*-*ABL1*-positive as well as Ph-like ALL [15, 16].

Additional interphase FISH experiments were performed to detect the *IKZF1*–*TYW1* fusion gene using a home-made dual color dual fusion probe. The probes were made from commercial BACs which were purchased from BACPAC Resources Center (https://bacpacresources.org). The probe for the *IKZF1* gene was constructed from a pool of the clones RP11-813K3 (Accesion number AC020743; Position: chr7: 50157413–50339940) and RP11-95E2 (Accesion number AC018705; chr7: 50475184–50648153) and was labeled red (Fig. 3c, d). The probe for the *TYW1* gene was constructed from a pool of the clones RP11-458F8 (Accesion number AC073335; Position: chr7: 66297269–66454983) and RP11-166O4 (Accesion number AC006480; Position: Chr7: 66699524–66859231) and was labeled green (Fig. 3c, e).

FISH analysis showed double fusion signals in 91 out of 100 examined interphase nuclei from white blood cells suggesting an *IKZF1*–*TYW1* fusion gene (Fig. 3f).

## Discussion

We present here a case of pediatric leukemia with a normal karyotype, 46,XX, but with multiple hidden aberrations identified using a combination of aCGH, RNA sequencing, and FISH methodologies. The initial FISH analysis with a *PDGFRB* breakapart probe showed loss of the distal part of the probe suggesting a genetic rearrangement of the *PDGFRB* gene. Because loss of the distal part of the probe was seen in interphase nuclei and not on metaphase spreads, we concluded that the normal karyotype was from non-leukemic cells whereas the leukemic cells did not divide in vitro. Subsequent analyses by aCGH revealed submicroscopic deletions within the *TCRB* (7q34), *PAX5* (9p13), *DMBT1* (10q26.13), 14q11.2 (*TRAC*), and *IGH* (14q32) loci, as well as loss of a 30 Mb stretch which started with *PDGFRB* (5q32) and finished in the *CANX* locus (5q35.3). The aCGH results were in agreement with previous findings of submicroscopic deletions found at high frequencies in ALL [2, 6–8]. Many *PAX5* deletions were reported in *BCR*-*ABL1*-positive and Ph-like ALL [6, 7, 17]. *DMBT1* deletions were reported in tumors of the central nervous system but this gene’s role, if any, in ALL development remains unknown [18, 19]. Finally, concomitant deletions within the immune loci *TCR* (alpha/delta and gamma) and *IGH*, which are often accompanied by deletions within *IKZF1*, were shown to be associated with lymphoid blast transformation of chronic myeloid leukemia [20]. The deletion of 30 Mb in 5q32q35.3, found by aCGH and FISH using a *PDGFRB* breakapart probe, started within *PDGFRB* (5q32), indicating the presence of a *PDGFRB*-fusion gene which we set out to find using RNA sequencing. Combining RNA sequencing, PCR/Sanger sequencing, and FISH methodologies we found and verified two fusion transcripts: The first was a novel in-frame *GTF2I*–*PDGFRB* fusion transcript. Due to the extensive homology of the *GTF2I* gene with two pseudogenes, *GTF2IP1* and *GTF2IP4*, neither RNA sequencing nor the subsequent PCR/Sanger sequencing analyses could verify the precise fusion. FISH analyses based on hybridization with specific probes for the *GTF2I* and *PDGFRB* genes helped to identify the fusion as a *GTF2I*–*PDGFRB*, however. Similar to the FISH experiments with the *PDGFRB* breakapart probe, the fusion signal suggesting a *GTF2I*–*PDGFRB* hybrid gene was seen on interphase nuclei but not on the examined metaphase spreads. In all likelihood, the leukemic cells did not divide in vitro.

Based on the reference sequences NM_032999.3/NP_127492.1 (*GTF2I*) and NM_002609.3/NP_002600.1 (*PDGFRB*), the *GTF2I*–*PDGFRB* fusion gene would code for a putative 1671 amino-acid-residues (aa) chimeric protein containing almost the entire GTF2I protein (979 out of 998 aa) and the part of PDGFRB (aa 415 to 1106 in NP_002600.1) which contains the catalytic domain of the protein tyrosine kinase. The putative *GTF2I*–*PDGFRB* fusion protein seems to be an abnormal protein tyrosine kinase in a similar way to what has been seen with other PDGFRB fusion proteins [21].

Hematologic neoplasms with *PDGFRB*-fusion genes can be treated with tyrosine kinase inhibitors [22–24]. Whether this applies also to the present case, remains unknown since complete remission was obtained using standard high-risk ALL treatment.

The second novel fusion transcript was an out-of-frame *IKZF1*–*TYW1* which would code for a truncated 159 aa IKZF1 protein containing the first 140 aa of IKZF1 (NP_006051) and 19 aa resulting from the fusion with TYW1. The *IKZF1* and *TYW1* genes were mapped on chromosome subbands 7p12.2 and 7q11.21. Thus, *IKZF1*–*TYW1* was most probably the product of a 7p12.2/7q11.21 recombination event: maybe an inv(7)(p12q11) or a t(7;7)(p12;q11) rearrangement. Fusions of *IKZF1* with various partner genes have been reported before in ALL [25, 26]. However, to the best of our knowledge, this is the first time that an *IKZF1*–*TYW1* fusion transcript is reported. Alterations of *IKZF1* (often deletions) are known to be strongly associated with *BCR*-*ABL1*-positive and Ph-like ALL [15, 16].

## Conclusions

The examined pediatric leukemia was a Ph-like ALL which carried a novel *GTF2I*–*PDGFRB* fusion gene, a novel *IKZF1*–*TYW1* fusion gene, and submicroscopic deletions on chromosome bands 5q32q35.3, 7q34 (within *TCRB*), 9p13 (*PAX5*), 10q26.13 (*DMBT1*), and 14q32.33 (within the *IGH* locus).

## Data Availability

All available data are included in the manuscript and its figures.

## Abbreviations

aCGH: array comparative genomic hybridization
ALL: acute lymphoblastic leukemia
BAC: bacterial artificial chromosome
FISH: fluorescence in situ hybridization
RT-PCR: reverse transcription-polymerase chain reaction
SNP: single nucleotide polymorphism
